# Variation in the Circulating Levels of Retinoic Acid and Type 2 Diabetes: Relationships with Glycemic Control Indices, Disease Treatment and Complications

**DOI:** 10.3390/diagnostics16091359

**Published:** 2026-04-30

**Authors:** Aseel A. Alsaidan, Basil M. Alomair, Abdulrahman H. Almaeen, Jumanah M. Q. Aldirbas, Bdour R. S. Alanazi, Raghad H. S. Algaed, Alanoud F. B. Alruwaili, Nouf M. S. Alruwaili, Duna F. A. Aljbab, Lama A. A. Alenzi, Razan S. S. Albalawi, Alaa A. Mohamed, Tarek H. EL-Metwally

**Affiliations:** 1 Department of Family and Community Medicine, College of Medicine, Jouf University, Sakaka 72388, Saudi Arabia; aaalsaidan@ju.edu.sa; 2 Department of Internal Medicine, College of Medicine, Jouf University, Sakaka 72388, Saudi Arabia; bmalomair@ju.edu.sa; 3 Department of Pathology, College of Medicine, Jouf University, Sakaka 72388, Saudi Arabia; alaaali@ju.edu.sa; 4 College of Medicine, Jouf University, Sakaka 72388, Saudi Arabia; jmoonh29@gmail.com (J.M.Q.A.); bdouranazi@gmail.com (B.R.S.A.); raghadhamdan36@gmail.com (R.H.S.A.); fadelalanoud@gmail.com (A.F.B.A.); noufmajed.000@gmail.com (N.M.S.A.); dunaf20@gmail.com (D.F.A.A.); lolaa8989@hotmail.com (L.A.A.A.); rozyy774@gmail.com (R.S.S.A.); 5 Department of Medical Biochemistry, Faculty of Medicine, Beni-Suef University, Beni-Suef 62521, Egypt; 6 Department of Medical Biochemistry and Molecular Biology, Faculty of Medicine, Assiut University, Assiut 71526, Egypt

**Keywords:** type 2 diabetes, retinoic acid, complications, glycemic control, insulin resistance

## Abstract

**Background/Objectives:** Type 2 diabetes mellitus (T2-DM) is a continuing national and global health challenge. Retinoic acid (RA), the major transcription-regulating ligand, plays a critical role in energy metabolism, and pancreatic β-cell homeostasis. However, human data linking circulating RA levels to T2-DM and its clinical outcomes are sparse and inconsistent. In this ethically approved cross-sectional study of consented hospital-diagnosed adult T2-DM patients (*n* = 292) and matched healthy controls (*n* = 64), variation in plasma RA levels and its relationship with disease and patient characteristics were investigated. **Methods:** RA concentrations assayed via specific ELISA were related to glycemic control indices [fasting blood glucose (FBG) and HbA1c], the triglyceride–glucose ratio for insulin resistance (TyG-IR), treatment modalities, and complications derived from patients’ medical records. **Results:** RA concentrations were substantially lower in patients with T2-DM (mean ± SD 2.63 ± 1.54 ng/mL) than in controls (5.21 ± 4.3 ng/mL; *p* < 0.001). Within the diabetic cohort, RA was inversely correlated with indices of glycemic dysregulation and insulin resistance. Plasma RA exhibited strong discriminatory performance for distinguishing diabetic patients from healthy adults. Its AUC is 0.870 (*p* < 0.0001 and 95% CI = 0.832–0.902) with a sensitivity of 79.7% and a specificity of 81.3%, at an optimal cutoff of ≤3.061 ng/mL. **Conclusions:** Circulating RA is associated with metabolic perturbations that define T2-DM, and therefore is promising as a clinically useful biomarker. It may reflect pathophysiological processes linking nutrient signaling, energy handling and β-cell function in T2-DM that merit further evaluation.

## 1. Introduction

Type 2 diabetes mellitus (T2-DM) is a significant global and national health challenge, where Saudi Arabia has one of the worst prevalence (~30%) and complication scenarios. T2-DM comprises chronic insulin resistance and progressive pancreatic β-cell dysfunction, transdifferentiation, and/or dedifferentiation as a consequence of the unsettled hyperglycemia in a milieu of high body mass index (BMI) and low-grade inflammation. Considering its clinical presentation and disease progression, T2-DM heterogeneity stems from its multifactorial polygenic–life style–environment interaction causation [1,2,3]. Despite improvements in therapy and lifestyle changes, its incidence is increasing at an alarming rate, which has fueled a shift toward disease prevention through the identification of high-risk groups and factors [4].

Vitamin A (all-trans retinol), an essential micronutrient, functions mainly through its transcriptionally active endogenous form, all-trans retinoic acid (atRA). RA controls stem cell homeostasis, cell differentiation, and cell metabolism, pre- and post-natal. Intracellular RA is active at nanomolar concentrations that require spatiotemporal precise homeostatic regulation. Its inverted J-shaped hormetic dose–response curve implies a transition from beneficial to toxic as concentrations increase [5,6]. With retinol availability, the synthesis of atRA by the sequential action of retinol and retinal dehydrogenases is balanced with its self-controlled clearance by cytochrome (CYP) 450/CYP26 RA hydroxylases. Both systems, along with binding proteins and receptors, are expressed in various cell types, including the pancreas, adipocytes, muscles and immune cells, with major immunomodulatory activity [7,8,9]. In addition to its nongenomic actions, RA functions mainly as a ligand for its transcription-regulating nuclear retinoid receptors RARαβ and γ and RXRαβ and γ and modulates the action of other type II nuclear receptors, namely, peroxisome proliferator-activated receptors (PPARs) β/δ. Mutations and risk variants in retinol/atRA carrier/binding and receptor proteins and metabolizing enzymes are linked to susceptibility to and severity of metabolic, degenerative and inflammatory diseases, including diabetes. Notably, proteins implicated in RA homeostasis have metabolic actions that are independent of their roles in vitamin A physiology [10,11,12,13,14]. For example, aldehyde dehydrogenase 1A1 and 1A3 are signature genes connected to pancreatic β-cell dedifferentiation/transdifferentiation into α-cells and negatively correlate with insulin vs. glucagon expression [15]. Loss of retinol dehydrogenase 1 causes obesity by disrupting the adaptive role of RA to fast, decreasing brown adipose lipolysis and mitochondrial oxidative phosphorylation and thermogenesis, and enhancing lipid storage [16].

There are opposing reciprocal regulatory interactions between insulin and RA signaling and its synthesis/catabolism systems. Blockage of the insulin gene in diabetic transgenic pigs leads to a ~2.5-fold increase in atRA concentration, which is correlated with increases in retinol dehydrogenase 16 activity [17]. Loss of pancreatic β-cell mass/dysfunction is orchestrated mainly by hyperglycemia-induced inhibition of the expression and action of forkhead box-O1 (FoxO1), the major transcription regulator of energy metabolism. During fasting, glucagon and cortisol are inactivated, whereas the fed state and insulin activate FoxO1 to control the level of RA, which, in turn, modulates FoxO-1 activity [18,19,20,21]. This gives insulin a fed-sate upper hand, whereas RA works differently in the fasting state. The liver glycogen and retinol contents reciprocally correlate with each other. RA and retinol modulate the development of obesity and its comorbidities: insulin resistance, T2-DM and its complications, hepatic steatosis, and cardiovascular disease. The signaling pathways of insulin and RA transcriptionally regulate hepatic glucose and lipid metabolism, pancreatic β-cell functions, and glycemic control [7,22].

Dietary intake and circulating levels of retinol are pertinent to the development of DM, as RA promotes pancreas development and β-cell function, maintains glucose homeostasis, regulates pancreatic innate immune responses, and controls the differentiation/transdifferentiation of pancreatic stem cells [8]. While the link between the development of T1-DM and reductions in blood retinol levels is well established, the mutual impacts of T2-DM and vitamin intake, its blood level, and the resulting level of retinoic acid on each other appear to be uncertain [23,24,25]. Clinical studies conducted to assess the pathogenic and/or biomarker potential of changes in the circulating levels of RA in T2-DM patients and its complications are not only insufficient globally and nationally but also contradictory. Whether the development of insulin resistance and onset of T2-DM affect retinoids homeostasis and signaling, and whether alterations in retinoids metabolism and signaling facilitate or prevent T2-DM and its complications require future scrutinization [13,22,26,27,28,29,30,31].

On the basis of our own experience and the findings of previous studies, we anticipated a significant reduction in plasma levels of RA in diabetic patients, correlating with worse glycemic control and complications. With a cross-sectional design, we aimed to investigate the associations between the changes in the quantitatively assayed plasma levels of RA and the glycemic control biomarkers, complications and treatment of T2-DM. We also analyzed the biomarker ability of RA to differentiate the disease and its complications for early metabolic risk detection.

## 2. Materials and Methods

### 2.1. Study Design and Setting

A biomarker-based cross-sectional study was conducted at the diabetes outpatient clinics of Prince Muteb General Hospital and King Abdulaziz Specialized Hospital in Sakaka and Domat Al-Jandal General Hospital in Domat Al-Jandal, Al-Jouf Province, Saudi Arabia. We assessed the associations between circulating RA levels and various clinical parameters, including glycemic control indices and complication profiles in patients with T2-DM at a single point in time.

### 2.2. Study Participants and Sampling

The study population comprised two distinct adult groups (≥18 years) recruited concurrently to provide a comparative baseline for the analysis of RA in clinically confirmed hospital-diagnosed T2-DM patients and healthy controls with no history of diabetes or prediabetes. They are nonsmokers and alcohol-abstinent. The inclusion of the control group is to establish a reference range for physiological RA levels, as a standard methodological feature of cross-sectional biomarker studies, essential for: (1) establishing a normative baseline by defining the expected range of RA levels in a nondiabetic, metabolically healthy population; (2) enabling direct comparative analysis to quantify the degree of any RA dysregulation present in the T2-DM cohort at the same point in time; and (3) validating the discriminatory power through receiver operating characteristic (ROC) curve analysis for the assessment of RA’s ability to distinguish between diabetic and nondiabetic states.

Healthy controls were recruited from among the companions (family members and spouses) of patients to ensure broad socioeconomic similarity. They were selected to be comparable to the patient group in terms of age, sex, and BMI to minimize the potential confounding effects of these variables on RA levels. A consecutive sampling method was employed for both groups until the predetermined sample size was reached. The sample size calculated via Calculator.net (https://www.calculator.net/sample-size-calculator.html?type=1&cl=95&ci=5&pp=25&ps=400000&x=68&y=16 (accessed on 1 June 2023)) was determined to be 288 cases, with a margin error of 5%, a reported reduction in RA levels of >25% [26,27,28,32,33], and an Al-Jouf population of ~400,000. The final sample of informed and consented adults comprised 292 T2-DM patients and 64 healthy controls of both sexes. They were enrolled by direct contact after the ethical approval of this study by the Permanent Research Ethics Committee of Jouf University, Sakaka, Saudi Arabia (approval No. 9-09-44 on 19 June 2023). This study adhered to the tenets of the 2024-revised Declaration of Helsinki. We excluded participants receiving vitamin A supplementation or RA treatment in the past 3 months. Participants on strong CYP450-inducing drugs such as phenytoin, carbamazepine and valproate, and those on CYP450 inhibitors such as imidazole antifungal drugs were excluded. We also excluded patients with T1-DM, immobility, chronic kidney and liver failure, autoimmune diseases, pregnancy, all types of cancer, inflammatory conditions unrelated to T2-DM, and aggressive complications (such as diabetic foot and gangrene).

### 2.3. Investigations and Data Collection

The data collected included age, sex, disease duration, complication type [complication-free, microvascular (neuropathy, ophthalmopathy or nephropathy) and macrovascular (cardiac and stroke), type of current treatment (naïve, diet control, metformin, other hypoglycemic ± insulin), and routine laboratory investigations [fasting lipid profile; triglycerides, and total, low and high density lipoprotein cholesterol (LDL-C and HDL-C), glucose, hemoglobin A1c (HbA1c), and total while blood cell count (WBCs)]. The triglyceride–glucose insulin resistance index (TyG-IR), a noninsulin-based surrogate marker of insulin resistance, was calculated as Ln [(triglycerides, mg/dL × glucose, mg/dL)/2] [34].

Overnight fasting peripheral blood samples were aseptically collected on EDTA from the antecubital vein, centrifuged for 20 min at 4 °C and 1000× *g*, and aliquots were stored in brown tubes at −60 °C until batch-assayed. Circulating RA was quantitatively measured, in duplicates, using a specific quantitative ELISA as instructed (cat No. SL3696Hu; Sunlong Biotech Co., Ltd., Hangzhou, Zhejiang, China). The inter-assay coefficient of variation (CV) was <12%, and the intra-assay CV was <10%, with a limit of detection of 0.312 ng/mL. All procedures were carried out under dimmed yellow light to avoid light-induced destruction of RA [28].

### 2.4. Statistical Analysis Plan

The data were analyzed via statistical software (e.g., SPSS Statistics, version 27.0). Continuous variables are presented as the means ± standard deviations (SDs) if normally distributed or medians (ranges) if not normally distributed. Normality was assessed via the Shapiro–Wilk test. Categorical variables are presented as frequencies and percentages. Independent-sample *t*-tests or Mann–Whitney U tests for nonparametric data were used to compare RA levels and other continuous variables between the T2-DM and control groups. One-way ANOVA or the Kruskal–Wallis test was used for comparisons across multiple T2-DM subgroups (e.g., by complication type). The relationships between RA levels and continuous clinical variables (e.g., HbA1c and diabetes duration) were assessed via Spearman’s correlation coefficients. A receiver operating characteristic (ROC) curve was generated to evaluate the sensitivity and specificity of RA levels for distinguishing T2-DM patients from healthy controls, and the area under the curve (AUC) was calculated as a measure of the discriminatory power. A *p* value of <0.05 was considered statistically significant for all tests.

## 3. Results

A total of 292 patients were included, with a mean age of 55.7 years and a mean BMI of 31.5 kg/m^2^. Females represented 63.0% of the sample. The average disease duration was approximately 10.5 years. Clinical complications were observed in 33.9% (*n* = 99) of the subjects, primarily driven by microvascular issues such as ophthalmopathy and neuropathy. Only a small fraction of the population (6.5%) was managed by diet alone, whereas the remainder required pharmacological intervention, most notably metformin or insulin (Table 1).

The biochemical profiles of the cases and controls are presented in Table 2. Patients exhibited significantly deregulated glycemic parameters, as evidenced by markedly higher HbA1c, FBG, and TyG-IR indices (all *p* < 0.001) compared with controls. The diabetic cohort also presented a significantly more atherogenic lipid profile; specifically, triglycerides and LDL-C levels were significantly elevated in cases compared to controls (1.8 ± 0.9 vs. 1.0 ± 0.3 mM/L and 2.7 ± 1.1 vs. 2.2 ± 0.3 mM/L, respectively; *p* < 0.001), while HDL-C levels were significantly lower (1.3 ± 0.6 vs. 1.8 ± 0.2 mM/L, *p* < 0.001). Interestingly, total and LDL-C cholesterol levels did not differ significantly between the two groups (*p* = 0.964 and *p* = 0.464, respectively). Patients had significantly greater, although clinically normal, WBC counts than controls did (*p* = 0.004). The plasma RA levels were significantly lower in the cases than in the controls (2.63 ± 1.54 vs. 5.21 ± 4.3 ng/mL, *p* < 0.001), with a mean difference of approximately 2.58 ng/mL.

Table 3 presents patients stratified by the presence or absence of diabetic complications to assess the associations with patients’ and disease characteristics. The associations with patient age (*p* < 0.001), disease duration (*p* < 0.001), fasting blood glucose (*p* = 0.013) and treatment with insulin ± other treatments (*p* < 0.001) were significant. Fractionation of disease duration into ≤3, 3–5, and >5 years, or ≤10 and >10 years revealed a significant association (*p* = 0.025).

Comparisons of plasma RA levels across various demographic (gender) and clinical (complications, nature of treatment, and disease duration) subgroups of type 2 diabetic patients revealed no significant differences.

**Figure 1:**
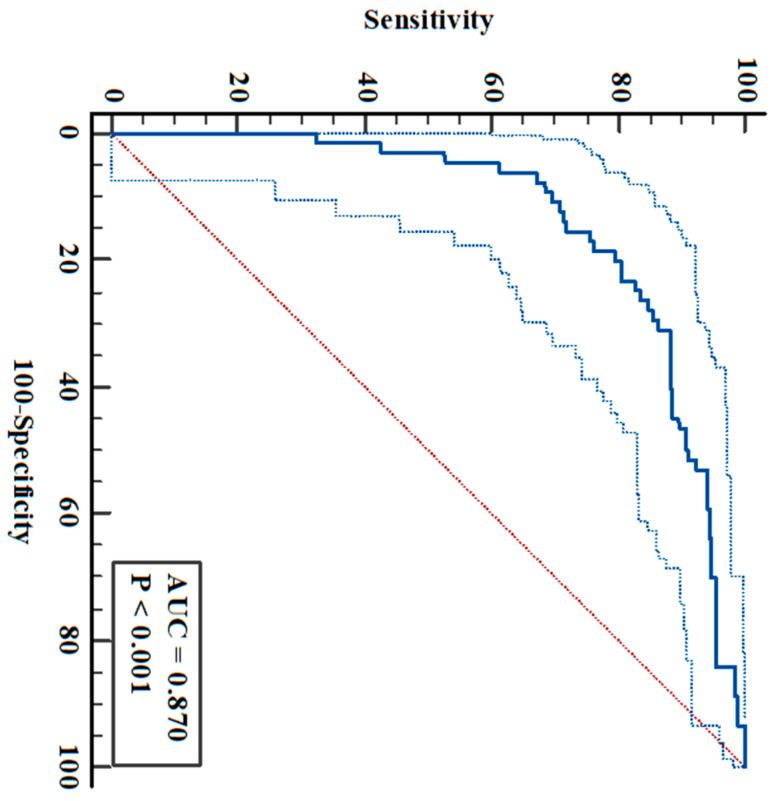
Receiver operating characteristic (ROC) curve for plasma retinoic acid levels (ng/mL). It exhibited excellent discriminatory power between type 2 diabetic patients (*n* = 292) and healthy controls (*n* = 64). AUC = area under the curve at 95% CI: 0.832–0.902, *p* < 0.0001.

The diagnostic utility of plasma RA was evaluated via receiver operating characteristic (ROC) curve analysis. RA exhibited excellent discriminatory power, with an area under the curve (AUC) of 0.870 (95% CI: 0.832–0.902; *p* < 0.0001). At the optimal cutoff value of ≤3.061 ng/mL (associated criterion), as determined by the Youden index (J = 0.609), the test demonstrated a sensitivity of 79.68% and a specificity of 81.25% (Figure 1).

The interrelationships between key variables within patients were assessed via Pearson’s correlation analysis (Table 4). Strong positive correlations were observed among the core glycemic indices: the TyG-IR index vs. each of fasting blood glucose (FBG, r = 0.719, *p* < 0.001) and HbA1c (r = 0.643, *p* < 0.001). The TyG-IR index also showed a strong positive correlation with its components; plasma triglycerides and glucose (r = 0.799 and 0.702, *p* < 0.001, respectively). Notably, there was a significant inverse correlation between RA levels and both TyG-IR (r = −0.332, *p* < 0.001) and HDL-C (r = −0.427, *p* < 0.001) levels. Conversely, HDL-C was inversely correlated with all the other glycemic parameters. Age showed a weak-to-moderate positive correlation with TyG-IR, blood glucose, and HbA1c.

## 4. Discussion

In this cross-sectional study of adult patients with T2-DM and matched healthy controls, circulating RA concentrations were markedly lower in the diabetic cohort than in the control cohort. Plasma RA demonstrated excellent diagnostic discrimination for T2-DM, with an AUC of 0.870 and an optimal cutoff of ≤3.061 ng/mL, reflecting strong sensitivity and specificity. Within the diabetic group, RA was inversely correlated with indices of glycemic dysregulation and insulin resistance (FBG, HbA1c, and TyG-IR). These findings suggest that alterations in circulating RA are closely linked to the metabolic dysregulation characteristic of diabetes and underscore its potential pathogenic role. Alterations in RA homeostasis likely reflect and may contribute to pathophysiological processes linking nutrient signaling, lipid homeostasis and β-cell function in T2-DM disease progression, as previously stated [7,22,26,28,29]. These findings indicate that RA merits further evaluation as a diagnostic and possibly prognostic biomarker when integrated with routine metabolic panels or risk stratification algorithms.

The reciprocal regulation between insulin signaling and RA biosynthesis/clearance, whereby fasting states promote RA synthesis and insulin/refeeding suppress it, provides a temporal framework for how RA concentrations are related to energy status and glucose homeostasis. The milieu of chronic over-nutrition, persistent hyperinsulinemia and insulin resistance could downregulate hepatic RA production and/or accelerate RA clearance. This produces lower steady-state circulating RA levels as we have documented. This blunts the RA-dependent protective programs, e.g., mitochondrial fatty acid oxidation and β-cell maintenance, exacerbating glycemic deterioration [19,20,21,35,36,37,38].

The observed reduction in circulating RA among patients with T2-DM aligns with previous reports of impaired retinoid metabolism in metabolic disorders [27,28,29,31]. The magnitude of difference and high diagnostic accuracy suggest that RA could complement existing glycemic and lipid markers for clinical risk assessment [28]. In fasting states, glucagon and cortisol induce hepatic RA synthesis through the activation of retinol dehydrogenases, whereas refeeding and insulin suppress RA via FoxO1 inhibition and the upregulation of CYP26-mediated RA clearance [7,19,20]. Chronic hyperinsulinemia in T2-DM patients could therefore reduce circulating RA concentrations and blunt its modulatory effects on energy homeostasis. Experimental studies showed that RA promotes lipid oxidation, enhances mitochondrial function, and supports pancreatic β-cell differentiation and survival [6,35,36,37,39]. The inverse associations of RA with HbA1c and TyG-IR reinforce the concept that RA homeostasis reflects core aspects of glucose–lipid interactions and insulin sensitivity. Reduced RA levels may thus represent both a consequence of insulin resistance and a contributing factor to its amplification [7,22].

Our findings confirm and extend those of previous human studies showing that lower RA levels are associated with poor glycemic control and increased metabolic risk. Liu and colleagues [33] demonstrated an inverse relationship between serum RA and incident metabolic syndrome, independent of adiposity and HOMA-IR. Similarly, Morgenstern et al. [28] reported that poorly controlled T2-DM patients presented markedly reduced plasma atRA levels, which were inversely correlated with triglycerides, LDL-C, and BMI, but positively correlated with HDL-C. However, the dyslipidemic effect of systemically used RA owing to its repressive effect on cholesterol 7α-hydroxylase, as the main cholesterol disposal venue into bile acids could complicate the picture [40,41]. Han et al. [29] reported that serum retinal and RA predict the development of T2-DM among subjects with impaired fasting glucose, emphasizing its prognostic value. Together with our data, RA deficiency may reflect a metabolically adverse phenotype. However, not all previous findings have been consistent. A reduced RA level in T2DM patients was positively correlating with HbA1c [42]. The marginal RA differences and/or paradoxical associations within specific contexts are likely due to variations in assay sensitivity, dietary vitamin A intake, or disease severity [8,31,43,44]. The hormetic nature of RA action, which is beneficial at physiological concentrations but detrimental at pharmacologic levels, complicates interpretation and underscores the importance of studying endogenous RA within its narrow physiological window [5,6].

The link between RA and T2-DM extends beyond correlation. RA exerts multifaceted control over glucose and lipid metabolism through the transcriptional regulation of enzymes such as glucokinase and pyruvate carboxykinase, and the modulation of sterol regulatory element binding protein 1 (SREBP1) and PPAR signaling [3,22,45]. Reduced RA signaling can impair β-cell maintenance, promote dedifferentiation, and diminish insulin secretory capacity [12,36]. It can also attenuate hepatic fatty acid oxidation and favor steatosis, thereby exacerbating systemic insulin resistance [7,39]. Animal and in vitro models support these mechanisms; inhibition of RA receptors or deficiency of retinol dehydrogenases leads to obesity, impaired thermogenesis, and glucose intolerance [16,35]. Conversely, the activation of RARβ2 or the administration of physiological levels of RA restores insulin sensitivity and reduces hepatic lipogenesis and oxidative stress in diabetic mice [46,47]. Hence, our observation of low circulating RA in human T2-DM patients likely reflects impaired retinoid homeostasis at both the systemic and cellular levels, contributing to the metabolic disturbances characteristic of diabetes.

Clinically, plasma RA showed potential diagnostic and stratification biomarker ability for T2-DM that could complement existing screening algorithms or serve as an adjunct marker for early metabolic risk detection. Future longitudinal studies should assess whether baseline RA can predict incident diabetes or the progression of complications [26,29]. Restoring the physiological RA signaling, particularly during fasting, whether through the modulation of dietary vitamin A, receptor agonists, enhancers of endogenous synthesis and/or protection from being catabolized, could improve metabolic resilience [22]. However, as high-dose retinoids can induce dyslipidemia and insulin resistance [48,49], any clinical translation must emphasize precise dosing, timing, and receptor-selective strategies.

The importance of the integrated roles of retinoids and liver in glucose disposal and the prevention of diabetes is no surprise, as retinol is almost solely stored in hepatic stellate cells, and the liver is the major glucose-turning-over tissue. Retinol is also stored in adipocytes and pancreatic stellate cells. In addition to their effect on RA homeostasis, the gene expression levels of retinoid receptors vary in response to fasting vs. refeeding physiological changes. RA induces pyruvate carboxykinase and glucokinase expression and synergizes with insulin to increase glucokinase and SREBP1 (the central regulator of lipid metabolism) expression levels in primary hepatocytes and glycogenesis in muscles [22,35,39]. Our laboratory and other researchers have shown that the RA signaling pathway is not only important for the welfare of pancreatic β-cells, insulin secretion and action but also directly activates β-cell neogenesis and prevents apoptosis [36,39,50,51,52,53]. Indirectly, atRA orchestrates energy metabolism in the whole body through the induction of atRA-responsive fibroblast growth factor (FGF) 21 secretion from the liver, which controls appetite and restores glucose and lipid homeostasis in obesity-induced diabetes [36,37,38]. RA ameliorates and/or its serum levels inversely correlate with the development of metabolic syndrome, obesity and DM complications in experimental and clinical settings, including micro- and macro-vascular diseases, and dermopathy/impaired wound healing [8,26,32,54,55,56,57].

The strengths of this study include its relatively large, well-characterized cohort, use of matched controls, rigorous exclusion of confounders, and adherence to pre-analytic and analytical protocols [28]. The inclusion of TyG-IR as a non-insulin-based surrogate marker of insulin resistance has allowed for deeper insight into metabolic correlations. This study contributes novel human data from a Middle Eastern population, an area underrepresented in metabolic biomarker research, and offers a potential translational bridge between basic retinoid biology and clinical diabetes management. Nevertheless, the cross-sectional design precludes establishing causality. Interventional and longitudinal studies are needed to determine temporal directionality. Single-point RA measurements may not fully capture fasting–feeding dynamics, although previous data indicate limited diurnal variation [58]. Moreover, ELISA-based quantification, while practical, lacks the specificity of LC-MS/MS for distinguishing RA isomers [59]. The inter-assay CV (<12%) is on the higher side of the limits. Dietary intake with vitamin A precursors and hepatic status, although unremarkable, were not measured and could contribute to inter-individual variability. Although these are not directly related, correlation of RA levels with the circulating retinol content, and the dissection of the iso-form distribution of the measured RA are worth investigating. A larger control group could have strengthened our findings.

## 5. Conclusions

Circulating RA is significantly reduced in T2-DM patients and is inversely correlated with glycemic control and insulin resistance indices. These findings reinforce the concept that RA is integral to metabolic regulation and has potential as a novel biomarker and therapeutic target in diabetes. If validated, RA measurement could become a clinically valuable addition to diabetes risk assessment and management frameworks, linking nutritional biochemistry with translational endocrinology. These observations support two complementary conclusions: (1) Circulating RA is associated with metabolic perturbations that define T2-DM and therefore has promise as a clinically useful biomarker. (2) Alterations in RA homeostasis likely reflect and may contribute to pathophysiological processes linking nutrient signaling, lipid handling and β-cell function in T2-DM. Future studies should employ mass spectrometric validation of RA species, explore tissue-specific RA metabolism, and test whether modulating retinoid pathways can improve glycemic control or mitigate diabetic complications [7,22,26].

## Figures and Tables

**Table 1:** The demographic, anthropometric and clinical characteristics of the study type 2 diabetic patients (*n* = 292).

Patients’/Disease’s Characteristics		n (%) Mean ± SD (Range) Median (Range)
Age, Years		55.7 ± 10.9 (23–99)
Gender	Female	184 (63.0)
	Male	108 (37.0)
Body mass index, kg/m^2^		31.5 ± 5.9 (20.0–58.6)
Disease duration, Years		10.5 ± 7.2 10.0 (0.1–32.0)
Presence of Complications	No	193 (66.1)
	Yes	99 (33.9)
Type of Complications	Ophthalmopathy	57 (19.5)
	Neuropathy	52 (17.8)
	Nephropathy	9 (3.1)
	Myocardial Infarction	9 (3.1)
	Stroke	5 (1.7)
Type of Treatment	Naïve/Diet Control	19 (6.5)
	Metformin	107 (36.6)
	Metformin ± Other hypoglycemics	66 (22.6)
	Insulin ± Hypoglycemics	100 (34.2)

**Table 2:** Comparison of the plasma metabolic and hematological parameters and retinoic acid levels between type 2 diabetic patients (*n* = 292) and healthy controls (*n* = 64).

Parameter	Cases	Controls	*p*
	Mean ± SD	Mean ± SD	
HbA1c, %	8.0 ± 1.9	5.5 ± 0.4	<0.001
FBG, mM/L	8.9 ± 3.7	5.0 ± 0.4	<0.001
TyG index	9.3 ± 0.6	7.4 ± 0.4	<0.001
TG, mM/L	1.8 ± 0.9	1.0 ± 0.3	<0.001
TC, mM/L	4.7 ± 1.2	4.7 ± 0.7	=0.964
HDL-C, mM/L	1.3 ± 0.6	1.8 ± 0.2	<0.001
LDL-C, mM/L	2.7 ± 1.1	2.2 ± 0.3	<0.001
WBCs, ×10^3^/µL	7.6 ± 1.9	6.9 ± 1.6	=0.004
RA, ng/mL [Median (IQR)]	2.4 (1.9–2.9)	3.9 (3.2–4.9)	<0.001

*p* < 0.05 was considered statistically significant (independent *t* test). SD = standard deviation, FBG = fasting blood glucose, HbA1c = % glycated hemoglobin, TyG-IR = triglyceride–glucose insulin resistance index, TG = fasting blood triglycerides, TC, HDL-C and LDL-C = fasting total, HDL-cholesterol and LDL-cholesterol, WBCs = total leukocyte count, and RA = retinoic acid.

**Table 3:** Type 2 diabetic patients’ and disease’s characteristics stratified according to the presence (*n* = 99) or absence (*n* = 193) of complications.

Variables			Complications		*p*
			No (193)	Yes (99)	
			Mean ± SD n (%)	Mean ± SD n (%)	
Gender	Female		123 (66.8)	61 (33.2)	=0.723
	Male		70 (64.8)	38 (35.2)	
Age, Years			53.9 ± 11.4	59.3 ± 9.1	<0.001
BMI, kg/m^2^			31.7 ± 6.2	31.3 ± 5.7	=0.609
Disease duration, Years	All		9.4 ± 6.5	12.8 ± 7.8	<0.001
	≤3		39 (81.3)	9 (18.8)	=0.025
	>3–5		21 (72.4)	8 (27.6)	
	>5		131 (61.5)	82 (38.5)	
	≤10		128 (70.7)	53 (29.3)	=0.025
	>10		63 (57.8)	46 (42.2)	
HbA1c, %			7.8 ± 1.7	8.2 ± 2.1	=0.080
FBG, mM/L			8.5 ± 3.3	9.9 ± 4.4	=0.013
TyG, mM/L			9.3 ± 0.6	9.4 ± 0.7	=0.358
TG, mM/L			1.8 ± 0.9	1.8 ± 0.9	=0.704
TC, mM/L			4.7 ± 1.3	4.6 ± 1.2	=0.341
HDL-C, mM/L			1.3 ± 0.5	1.4 ± 0.7	=0.619
LDL-C, mM/L			4.1 ± 18.5	2.6 ± 1.0	=0.440
WBCs, ×10^3^/µL			7.7 ± 1.7	7.5 ± 2.2	=0.488
RA, ng/mL			2.7 ± 1.8	2.5 ± 1.0	=0.280
Treatment	Insulin-containing regimen	Yes	146 (76)	46 (24)	<0.001
		No	47 (47)	53 (53)	
	Metformin	Yes	89 (72.4)	34 (27.6)	=0.054
		No	104 (61.5)	65 (38.5)	
	Other hypoglycemics	Yes	136 (63.3)	79 (36.7)	=0.087
		No	57 (74)	20 (26)	

*p* < 0.05 was considered statistically significant for the Mann–Whitney test and the Kruskal–Wallis test. SD = standard deviation; FBG = fasting blood glucose; HbA1c = % glycated hemoglobin; TyG-IR = triglyceride–glucose insulin resistance index; TG = fasting blood triglyceride; TC, HDL-C and LDL-C = fasting total, HDL-cholesterol and LDL-cholesterol; WBCs = total leukocyte count.

**Table 4:** Interrelationships between key variables in type 2 diabetic patients (*n* = 292).

		Age	BMI	DD	HbA1c	FBG	TyG-IR	TG	TC	HDL-C	LDL-C	WBCs
BMI	r	0.093										
	*p*	0.081										
DD	r	0.358	0.074									
	*p*	<0.001	0.211									
HbA1c	r	0.251	0.157	0.055								
	*p*	<0.001	0.004	0.369								
FBG	r	0.299	0.167	0.130	0.709							
	*p*	<0.001	0.007	0.068	<0.001							
TyG-IR	r	0.342	0.191	0.034	0.698	0.817						
	*p*	<0.001	0.003	0.658	<0.001	<0.001						
TG	r	0.151	0.158	−0.037	0.334	0.426	0.799					
	*p*	0.006	0.004	0.554	<0.001	<0.001	<0.001					
TC	r	−0.162	−0.023	−0.206	−0.004	−0.040	0.047	0.126				
	*p*	0.004	0.681	0.001	0.945	0.545	0.488	0.026				
HDL-C	r	−0.236	−0.070	−0.051	−0.407	−0.492	−0.588	−0.392	0.225			
	*p*	<0.001	0.227	0.430	<0.001	<0.001	<0.001	<0.001	0.000			
LDL-C	r	−0.063	0.018	−0.202	0.072	0.154	0.221	0.181	0.708	0.017		
	*p*	0.262	0.750	0.001	0.213	0.017	0.001	0.001	<0.001	0.779		
WBCs	r	0.026	0.118	0.124	0.156	0.138	0.200	0.150	−0.077	−0.189	−0.095	
	*p*	0.627	0.028	0.037	0.005	0.025	0.002	0.007	0.174	0.001	0.092	
RA	r	−0.216	−0.094	0.046	−0.304	−0.297	−0.322	−0.103	0.105	0.273	0.054	−0.090
	*p*	<0.001	0.076	0.431	<0.001	<0.001	<0.001	0.064	0.064	<0.001	0.334	0.092

Spearman’s rank correlation analysis; *p* and r values are presented. BMI = body mass index, DD = disease duration, FBG = fasting blood glucose, HbA1c = % glycated hemoglobin, TyG-IR = triglyceride–glucose insulin resistance index, TG = triglycerides, TC, LDL-C and HDL-C = total, LDL- and HDL-cholesterol, WBCs = total leukocyte count, RA = retinoic acid.

## Data Availability

The major data supporting the findings of this study are presented in this article and the spreadsheet of the raw data is available from the corresponding author upon reasonable request and after proper local ethical approval.

## Abbreviations

The following abbreviations are used in this manuscript:

AUC	Area under the ROC curve
BMI	Body mass index
CI	Confidence interval
CYP	Cytochrome P
CV	Coefficient of variation
DD	Disease duration
ELISA	Enzyme-linked immunoassay
FBG	Fasting blood glucose
FGF21	Fibroblast growth factor
FoxO1	Forkhead box-O1
HbA1c	% Glycated hemoglobin
HDL-C	HDL-cholesterol
LC-MS/MS	Liquid chromatography–tandem mass spectrometry
LDL-C	LDL-cholesterol
PPAR	Peroxisome proliferator-activated receptors
RA	Retinoic acid
RAR	Retinoic acid receptor
ROC	Receiver operating characteristic
RXR	Retinoid X receptor
SD	Standard deviation
SREBP1	Sterol regulatory element binding protein 1
T2-DM	Type 2 diabetes mellitus
TGs	Triglycerides
TC	Total cholesterol
TyG-IR	Triglyceride–glucose insulin resistance index
WBCs	Total leukocyte count
